# Folcisteine Safeguards Maize Against Copper–Cadmium Stress by Boosting the Activity of Photosynthesis-Related Enzymes and Antioxidant Defense Systems, Mediating Ascorbate–Glutathione Pathways and Hormonal Regulation

**DOI:** 10.3390/ijms26188938

**Published:** 2025-09-13

**Authors:** Ling Dong, Meng Zhao, Jingwen Wei, Yiping Fu, Zihan Xu, Lihua Xie, Wanrong Gu, Yu Zhou

**Affiliations:** 1 College of Agriculture, Northeast Agricultural University, Harbin 150030, China; dongling@neau.edu.cn (L.D.); zmeng1116@163.com (M.Z.); wjw20030820@163.com (J.W.); s240301011@neau.edu.cn (Y.F.); a01220352@neau.edu.cn (Z.X.); 2 Crop Development Research Institute, Heilongjiang Academy of land Reclamation Sciences, Harbin 150030, China; xielh00@163.com

**Keywords:** maize, folcisteine, leaf photosynthesis, O_2_·^−^ and H_2_O_2_ metabolism, ascorbate-glutathione, hormone levels

## Abstract

With the rapid development of industry and agriculture, soil heavy metal pollution has become increasingly severe. Copper (Cu) and cadmium (Cd) often co-occur in soils, exerting combined stress on crops. As a major food and feed crop, maize was studied under CuCd stress to assess the mitigating effects of exogenous Folcisteine (NATCA). Two varieties with contrasting tolerance (Jiuyuan 15 and Longfuyu 6) were subjected to composite stress (80 mg·L^−1^ CuSO_4_ + 100 mg·L^−1^ CdCl_2_), with or without 20 mg·L^−1^ NATCA. The impacts on photosynthesis, reactive oxygen species (ROS) metabolism, the ascorbate–glutathione cycle, and endogenous hormones were investigated. The results showed that CuCd stress reduced the activities of RUBPCase and PEPCase, inhibiting CO_2_ fixation, while NATCA application enhanced their activities and improved photosynthetic efficiency. Stress also induced ROS accumulation (elevated O_2_·^−^ and H_2_O_2_) and elevated electrolyte leakage, whereas NATCA reduced oxidative damage and stabilized membrane integrity. Additionally, NATCA boosted both enzymatic and non-enzymatic antioxidant capacity in the ascorbate–glutathione cycle, improving ROS scavenging. Stress disrupted endogenous hormone balance, decreasing IAA, GA, and ZR, and increasing ABA. NATCA application restored hormone levels toward balance, promoting growth and enhancing tolerance to CuCd stress. These findings demonstrate NATCA’s role in improving maize resilience under heavy metal stress.

## 1. Introduction

Soil heavy metal contamination has a substantial impact on the physiological and biochemical processes of maize. Specifically, it can lead to a reduction in photosynthesis efficiency, affect the activities of various enzymes, disrupt the balance of endogenous hormones, and ultimately result in a decline in maize biomass and yields [1]. Maize, as a crop characterized by relatively low accumulation of heavy metals, plays a crucial role in the remediation of heavy metal-contaminated soils [2]. However, it has been observed that maize kernels tend to accumulate significant levels of cadmium (Cd) and copper (Cu) [3]. Analysis of the environmental monitoring data provided by the Ministry of Agriculture indicates that approximately one-fifth of China’s total cultivated land area is affected by heavy metal contamination. This contamination has led to an annual reduction of around 10 million tons in grain production [4]. Heavy metals are absorbed and accumulated by plants from the soil, and once they enter the food chain, they pose risks to the entire ecosystem. For instance, an investigation into the characteristics and health risks related to heavy metal contamination in cultivated soil within a small watershed in the black soil region of Northeast China revealed notable pollution levels of Cd, Hg, and Cu [5]. Such findings highlight the importance of understanding and addressing the issue of soil heavy metal contamination to safeguard agricultural productivity and ecological safety. Former studies also showed that the accumulation status and ecological risks of heavy metals in the surface black soil (0–20 cm) of the Haigou River small watershed in Heilongjiang Province were analyzed. The US EPA model was used to evaluate the health risks of soil heavy metal pollution. The results showed that the average content of heavy metal elements in cultivated soil in the Haigou River small watershed was 16.334 mg/kg for Cu and 0.213 mg/kg for Cd. The single factor pollution index, comprehensive pollution index, and land accumulation index all indicated that heavy metals in the soil of the study area had begun to accumulate and enrich [6]. In the context of soil pollution in China, heavy metal contamination often occurs as a complex pollution scenario, with copper–cadmium compound pollution being the most prevalent.

The main sources of heavy metals in maize farmland soil are industrial and agricultural production activities. Research has found that soil pollution in maize fields is inversely proportional to the distance between the fields and tailings [7,8]. Research has found that due to the discharge of waste from leather factories, the heavy metal content in the soil of nearby maize fields exceeds the standard [9]. The farmland soil around the smelting plant shows a clear trend of pollution and enrichment of Cd, Hg, Zn, Pb, and Cu, with an overall level of severe pollution. Maize is contaminated with Cd and Pb, with Cd and Pb exceeding the standard rates of 69% and 46%, respectively [10]. The pollution of Cu and Cd caused by sewage irrigation is becoming increasingly serious, and long-term accumulation of Cu and Cd can cause pollution to soil and maize [11]. In the sampling and monitoring of irrigation wastewater, farmland, and maize in northern Ningxia, China, it was found that the content of Pb, Cd, and Cu in the irrigation wastewater exceeded the standard, and the heavy metal content in the irrigated farmland soil and maize exceeded the standard [12]. Excessive use of steel slag in farmland leads to the accumulation of Cr content in the soil surface, causing heavy metal pollution in maize fields [13]. The application of solid waste compost from household garbage can also increase the level of heavy metal pollution in maize farmland soil [14].

After treatment with Cu and Cd, the synthesis of chlorophyll is hindered, reducing the activity of photosynthesis-related enzymes and thus decreasing the photosynthetic capacity of plants [15,16,17,18]. There are multiple reasons why cadmium stress leads to a decrease in chlorophyll content, including its effects on several enzymes and subcellular tissues [19,20,21,22]. Rubisco is a key enzyme in the C_3_ pathway, accounting for over 50% of plant soluble leaf protein [23]. The carbon assimilation process is completed by Rubisco, and most of the total carbon atoms present in the organism pass through Rubisco’s active sites [24]. In addition to catalyzing the reaction of carbon dioxide fixation, Rubisco can also catalyze the reaction of oxygen with 1,5-diphosphate ribulose to produce phosphoglycolic acid, which also triggers the photorespiration process [25]. Copper cadmium significantly inhibits Rubisco activity, ultimately reducing photosynthetic rate [26,27,28]. Cd treatment resulted in inhibition of leaf growth, as well as decreased photosynthetic parameters, transpiration, and stomatal conductance [29]. The efficiency of the PSII complex in absorbing and utilizing light energy decreases after Cu and Cd treatment [30]. Cd^2+^ enters the guard cells of leaves through Ca^2+^ channels, affecting the pathway of ABA and causing stomatal closure. Cadmium also affects the biosynthesis of the precursor of chlorophyll biosynthesis [31]. Cu and Cd stress cause significant damage to chloroplasts. After entering the cell, Cu and Cd can bind to thiol groups on proteins in chloroplasts, thereby inhibiting chlorophyll synthesis [32,33,34]. Therefore, the impact of Cu and Cd stress on the plant photosynthetic system is a holistic damage, including the absorption and transformation of light energy, photosynthetic enzymes, and chloroplast structure.

Under natural conditions, reactive oxygen species (ROS) are typically generated within organelles that possess electron transport chains or exhibit high oxidative metabolism rates. Among these organelles, peroxisomes and mitochondria are particularly prone to ROS production [35]. Copper (Cu) and cadmium (Cd), which are divalent cations, do not have the inherent ability to directly generate free radicals within plants. Nevertheless, numerous studies have documented the occurrence of ROS generation in plants subsequent to copper–cadmium stress [36]. Under physiological conditions, Cd^2+^ ions can substitute for metal ions on other proteins. This substitution process leads to an increase in the quantity of active antioxidant minerals as well as free reducing metal ions within cells. Subsequently, these free redox-active metals can directly augment the production of hydroxyl radicals through specific interactions [37]. Mitochondria serve as crucial sites for ROS production; however, the quantity of ROS generated under normal circumstances differs significantly from that under Cu and Cd stress conditions. In healthy tissues, the amount of free radicals produced by mitochondria is relatively low. In contrast, when subjected to stress, the production of ROS by mitochondria increases substantially. These elevated levels of ROS may trigger a series of detrimental consequences, including membrane lipid peroxidation, mitochondrial DNA breakage, impairment of ATP synthesis, mitochondrial damage, and ultimately, induction of cell apoptosis [38]. It has been demonstrated that Cd stress significantly elevates the relative electrical conductivity of both the aboveground and underground parts of two maize varieties during the germination stage. Moreover, as the concentration of Cd increases, the extent of membrane damage in each part shows a significant upward trend [39]. Additionally, research findings indicate that as the concentration of copper ions rises, the electrical conductivity and malondialdehyde (MDA) content of the extracellular exudate of petunias also increase significantly, thereby revealing a highly significant positive correlation between copper ions and both electrical conductivity and MDA [40].

In plants, endogenous hormones play a significant role in regulating plant resistance under stress conditions. Specifically, abscisic acid (ABA), indole-3-acetic acid (IAA), jasmonic acid (JA), and ethylene (ET) are of major importance in modulating plant defense and resistance responses to both biotic and abiotic stressors [41]. IAA is involved in multiple aspects of plant growth and development. It can regulate the development of plant embryos and, at low concentrations, promote the growth of plant roots, stems, leaves, and flowers, thereby exerting a crucial influence on plant sexual growth. Moreover, IAA can also interact with other hormonal pathways. It has been shown to inhibit the biosynthesis of cytokinins and activate the ABA signaling pathway as well as the brassinosteroid signaling pathway [42,43,44,45]. Similarly, zeatin riboside (ZR) at low concentrations can contribute to plant physiological processes. It can promote cell division and collaborate with auxin to facilitate the germination of callus tissue, enhance root development, regulate the construction of stem apical meristematic tissue, and ultimately strengthen plant resistance to adverse conditions. Gibberellic acid (GA), on the other hand, can promote seed germination, fruit development, and stem elongation [46]. Regarding ABA, its effect on seed germination under cadmium (Cd) stress is concentration-dependent. A low concentration of ABA can alleviate the toxic effects of Cd on seed germination, whereas a high concentration of ABA exacerbates the Cd toxicity and inhibits seed germination [47]. Numerous studies have demonstrated that within plants, each plant hormone does not act in isolation but rather needs to cooperate with other plant hormones to achieve complex and effective regulation in different growth stages, under various environmental conditions, and within specific tissues [48,49,50]. The coordinated regulation of both synergistic and antagonistic hormone effects, along with the regulation of hormone biosynthesis pathways, is crucial for plant resistance to adverse circumstances [51]. Under cadmium stress, the levels of endogenous hormones in plants can be altered, disrupting the hormonal balance. For instance, under high concentrations of Cd stress, the content of hydrogen peroxide (H_2_O_2_) increases, and H_2_O_2_ functions as a signaling molecule downstream of IAA, which consequently leads to a decrease in IAA content [52]. It is worth noting that many processes that impact crop performance and yield are governed by the balance of plant hormones, which is specific to particular organs or tissues. As the treatment time and concentration of Cd increase, the ratio of ABA to ZR gradually rises, and the antagonistic interaction between endogenous ABA and ZR under Cd stress might exert a protective effect against Cd toxicity [53]. Research findings have also indicated that under copper (Cu) stress, the ratio of GA to ABA in rapeseed leaves significantly decreases, and the ratios of IAA to ABA and ZR to ABA all exhibit a decreasing trend, with similar patterns in their changes [54]. Additionally, under Cu stress conditions, as the concentration of Cu^2+^ increases, the contents of ZR and GA in the leaves of grafted and self-rooted seedlings decline [55]. Furthermore, Cd treatment has been shown to inhibit the synthesis of endogenous hormones such as IAA, GA_3_, and cytokinin (CTK) in Chinese fescue, while promoting the synthesis of ABA [56].

Folcisteine (NATCA) is often used as an intermediate in pesticides and mixed with other substances to play a role in agricultural production. A plant growth regulator composition composed of hemiphyllin, inositol peptide, and potassium magnesium sulfate has specific functions of stimulating plant absorption of water and fertilizer, stabilizing the structure of large molecular substances, increasing the activity of metabolic enzymes in plants, regulating redox potential, increasing the content of amino acids in plants, and improving protein synthesis rate [57]. The plant growth regulator composition composed of hemifolin and auxin can act on agricultural production in the form of powder solubilizer, granulator, and ultra-low molecular spray, which is conducive to promoting fruit expansion and grain plumpness after passing away, improving root growth capacity and increasing yield [58]. A plant growth regulator combination composed of hemiphyllin and sodium salicylate can effectively improve the growth of crops such as wheat, rice, maize, soybeans, and peanuts under stress environments, enhance crop salt and alkali tolerance, and alleviate cell damage under crop stress [59]. In the soil environment, the chemical reaction process between the exogenous compound hemiphyllin (NATCA) and two heavy metals, namely copper and cadmium, plays a significant role. Through chelation, NATCA can modify the existing forms of copper and cadmium. Specifically, it can transform these heavy metals from their highly active and easily plant-absorbable forms into relatively stable forms with lower bioavailability. This transformation effectively alleviates the stress exerted on crop growth.

Research on heavy metal stress in maize has witnessed progress across multiple aspects. It has ranged from clarifying the impacts of stress on diverse aspects of maize growth to delving into tolerance mechanisms and seeking mitigation strategies. Nevertheless, numerous aspects still await further exploration. Notably, there has been a lack of reports regarding the utilization of exogenous hemiphyllin (NATCA) as a plant growth regulator in enhancing maize’s resistance to heavy metals [60]. In this study, maize varieties with varying sensitivities to heavy metals were selected as experimental materials. The aim was to comprehensively investigate the effects of exogenous NATCA on several key physiological processes in maize leaves under copper–cadmium compound stress. These processes encompassed the activities of key photosynthetic enzymes, active oxygen metabolism, the ascorbate–glutathione cycle, and hormone metabolism. By conducting comprehensive research that spans from theoretical mechanisms to practical applications, novel technological approaches can be unearthed. Such approaches are expected to enhance the production capacity of farmland that is polluted by copper–cadmium compounds in the field. Simultaneously, a robust theoretical and practical foundation can be established for the application of exogenous NATCA in the field. This, in turn, will contribute to promoting the sustainable utilization of polluted farmland and facilitating the green development of agriculture.

## 2. Results

### 2.1. Photosynthetic Key Enzymes Activity

**Figure 1:**
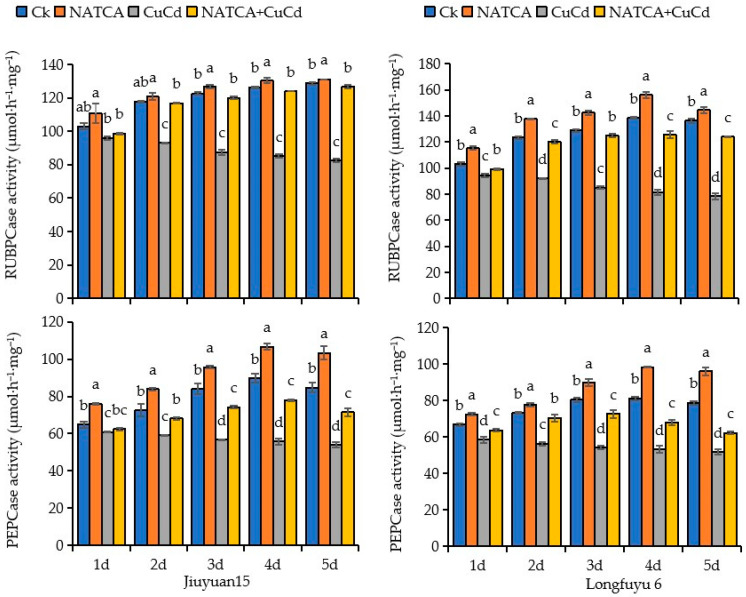
Effects of Folcisteine (NATCA) on photosynthetic key enzymes activity in maize seedlings roots under combined copper–cadmium stress. Ck represents 1/2 Hoagland nutrient solution; NATCA represents 1/2 Hoagland nutrient solution containing 20 mg·L^−1^ NATCA; CuCd represents 1/2 Hoagland nutrient solution containing 80 mg·L^−1^ CdCl_2_ and 100 mg·L^−1^ CuSO_4_; NATCA + CuCd represents 1/2 Hoagland nutrient solution containing 20 mg·L^−1^ NATCA, 80 mg·L^−1^ CdCl_2_, and 100 mg·L^−1^ CuSO_4_. Different letters within the same column indicate significant difference at 5% level.

As shown in Figure 1, compared with CK, the RUBPCase activity and PEPCase enzyme activity of maize seedling leaves treated with exogenous NATCA were significantly increased. At 5 days after treatment, the RUBPCase activity of Jiuyuan 15 reached its maximum value, which was 1.79% higher than that of CK; when processing for 4 days, the RUBPCase enzyme activity of Longfuyu 6 reached its maximum value, which was 12.70% higher than that of CK. During the 4 days of treatment, the PEPCase enzyme activity of Jiuyuan 15 and Longfuyu 6 reached their maximum values, which were 18.84% and 21.25% higher than the CK treatment, respectively. The activity of RUBPCase and PEPCase enzymes in maize seedling leaves after CuCd treatment decreased with increasing treatment time. Among them, the RUBPCase and PEPCase enzyme activities in the leaves of Longfuyu 6 seedlings showed the most significant decrease. Compared with CK, the RUBPCase enzyme activity in the leaves of Jiuyuan 15 decreased by 7.22%, 21.08%, 28.72%, 32.65%, and 35.88% after being treated with CuCd for 1–5 days; During the 5-day treatment with CuCd, the RUBPCase enzyme activity of Longfuyu 6 decreased by 8.98%, 25.43%, 33.96%, 41.22%, and 42.94%, respectively. Compared with CK, the PEPCase enzyme activity of Jiuyuan 15 decreased by 6.07%, 22.51%, 32.43%, 37.94%, and 36.41% after 1–5 days of CuCd treatment, respectively. The PEPCase enzyme activity of Longfuyu 6 decreased by 12.42%, 22.95%, 48.61%, 34.33%, and 34.32%, respectively, on days 1–5 of CuCd treatment. After treatment with NATCA + CuCd, the activity of RUBPCase and PEPCase enzymes in the leaves of two varieties increased significantly, which was higher than that of CuCd treatment. Compared with CuCd treatment, the RUBPCase enzyme activity of Jiuyuan 15 increased by 2.81%, 25.61%, 37.24%, 45.55%, and 53.43% after 1–5 days of treatment with NATCA + CuCd, respectively. Longfuyu 6 showed an increase in RUBPCase enzyme activity of 5.13%, 30.69%, 46.81%, 54.15%, and 57.71% in NATCA + CuCd treatment from day 1 to day 5, respectively. The PEPCase enzyme activity of Jiuyuan 15 increased by 2.51%, 15.29%, 30.97%, 39.96%, and 32.65% after 1–5 days of treatment with NATCA + CuCd, respectively. The PEPCase enzyme activity of Longfuyu 6 increased by 8.61%, 24.98%, 34.55%, 27.54%, and 20.49%, respectively, on days 1–5 after NATCA + CuCd treatment (Figure 1).

### 2.2. Superoxide Anion (O_2_·^−^)and Hydrogen Peroxide (H_2_O_2_)

**Figure 2:**
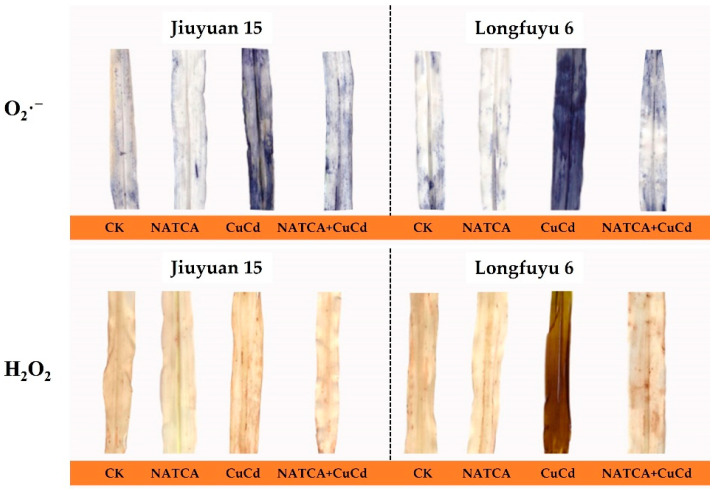
Effects of Folcisteine (NATCA) on detection of superoxide anion (O_2_·^−^) and hydrogen peroxide (H_2_O_2_) in the leaves of maize seedlings under combined copper–cadmium stress. Ck represents 1/2 Hoagland nutrient solution; NATCA represents 1/2 Hoagland nutrient solution containing 20 mg·L^−1^ NATCA; CuCd represents 1/2 Hoagland nutrient solution containing 80 mg·L^−1^ CdCl_2_ and 100 mg·L^−1^ CuSO_4_; NATCA + CuCd represents 1/2 Hoagland nutrient solution containing 20 mg·L^−1^ NATCA, 80 mg·L^−1^ CdCl_2_, and 100 mg·L^−1^ CuSO_4_. Different letters within the same column indicate significant difference at 5% level.

Maize seedlings subjected to CuCd stress produce a large amount of reactive oxygen species and cause disturbances in peroxide metabolism, including hydrogen peroxide (H_2_O_2_) and superoxide anion (O_2_·^−^). In DAB color development, DAB can react with peroxides to form a brownish substance that is insoluble in organic solvents such as water and alcohol. Using NBT staining, superoxide anions can react with nitrogen blue tetrazole to form blue methyl hydrazone. As shown in Figure 2, the distribution of brown spots on the leaves of maize seedlings represents the content and distribution of H_2_O_2_, while blue represents the content and distribution of O_2_·^−^. In situ detection of hydrogen peroxide and H_2_O_2_ in vivo tissue chemistry revealed that maize leaves treated with CuCd had the most brown spots. Compared with CuCd treatment, the maize leaves treated with NATCA + CuCd showed fewer brown spots, but more than those treated with NATCA. In situ detection of superoxide anion (O_2_·^−^) in live tissue chemistry showed that the maize leaves treated with CuCd had the most blue spots. Compared with CuCd treatment, the leaves of maize treated with NATCA + CuCd showed fewer blue spots, but more than those treated with NATCA. The content of hydrogen peroxide (H_2_O_2_) and superoxide anion (O_2_·^−^) in maize leaves treated with CuCd was high, and the content of hydrogen peroxide (H_2_O_2_) and superoxide anion (O_2_·^−^) in maize leaves decreased after NATCA treatment (Figure 2).

### 2.3. Electrolyte Leakage (EL)

**Figure 3:**
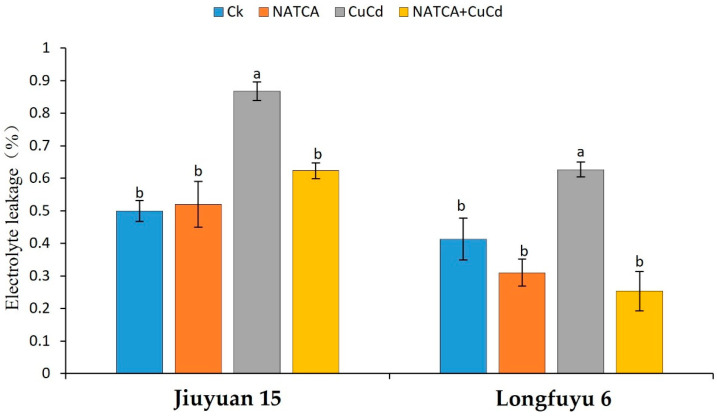
Effects of Folcisteine (NATCA) on electrolyte leakage (EL) in leaves of maize seedlings under combined copper–cadmium stress. Ck represents 1/2 Hoagland nutrient solution; NATCA represents 1/2 Hoagland nutrient solution containing 20 mg·L^−1^ NATCA; CuCd represents 1/2 Hoagland nutrient solution containing 80 mg·L^−1^ CdCl_2_ and 100 mg·L^−1^ CuSO_4_; NATCA + CuCd represents 1/2 Hoagland nutrient solution containing 20 mg·L^−1^ NATCA, 80 mg·L^−1^ CdCl_2_, and 100 mg·L^−1^ CuSO_4_. Different letters within the same column indicate significant difference at 5% level.

As shown in Figure 3, the electrolyte leakage rate of maize seedling leaves treated with CuCd increased, and Longfuyu 6 showed a more significant effect. Compared with CK, the electrolyte leakage rates of Jiuyuan 15 and Longfuyu 6 increased by 53.34% and 78.44%, respectively, after CuCd treatment. After CuCd treatment, the electrolyte leakage rate of Longfuyu 6 is 25.1% higher than that of Jiuyuan 15. The damage caused by CuCd stress to Longfuyu 6 is higher than that to Jiuyuan 15. Compared with CuCd treatment, the electrolyte leakage rates of Jiuyuan 15 and Longfuyu 6 were reduced by 18.70% and 69.36%, respectively, after NATCA + CuCd treatment. After NATCA treatment, the electrolyte leakage rate of the two varieties of maize decreased by 45.22% and 62.50% compared to CuCd. NATCA can alleviate CuCd stress in maize seedlings, and NCTCA has a more significant effect on the resistance of Longfuyu 6 to CuCd (Figure 3).

### 2.4. Activity of AsA–GSH Cycle Enzyme

**Figure 4:**
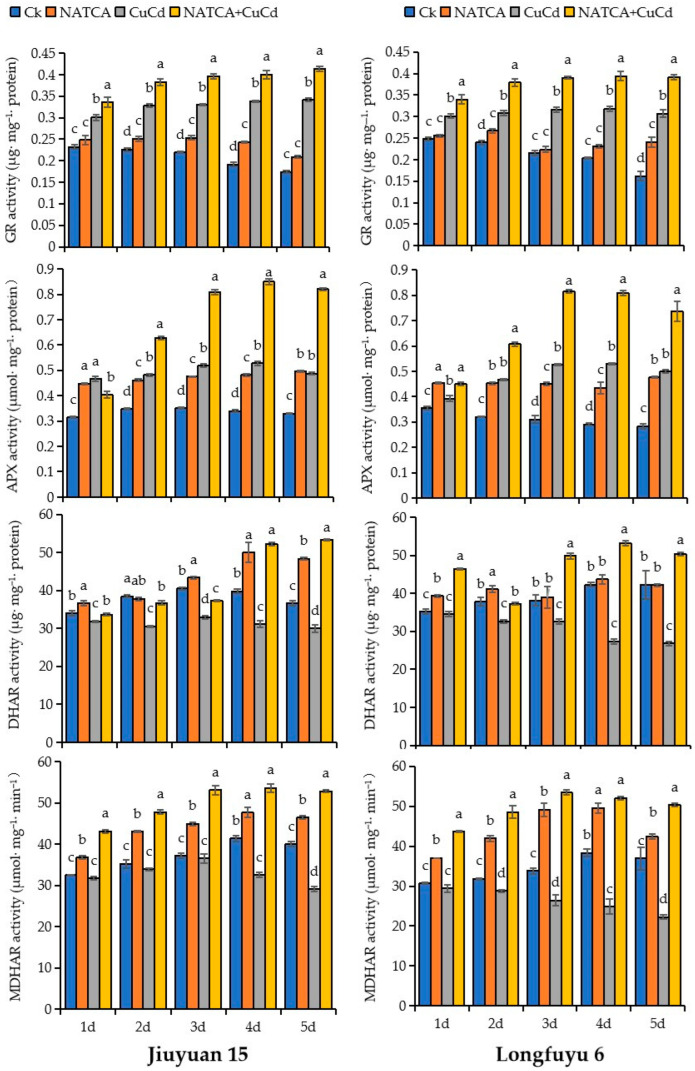
Effects of Folcisteine (NATCA) on the activity of ascorbate–glutathione cycle (AsA–GSH cycle) in maize seedling leaves under combined copper–cadmium stress. Ck represents 1/2 Hoagland nutrient solution; NATCA represents 1/2 Hoagland nutrient solution containing 20 mg·L^−1^ NATCA; CuCd represents 1/2 Hoagland nutrient solution containing 80 mg·L^−1^ CdCl_2_ and 100 mg·L^−1^ CuSO_4_; NATCA + CuCd represents 1/2 Hoagland nutrient solution containing 20 mg·L^−1^ NATCA, 80 mg·L^−1^ CdCl_2_, and 100 mg·L^−1^ CuSO_4_. Different letters within the same column indicate significant difference at 5% level.

The AsA GSH cycle can clear H_2_O_2_ produced under copper and cadmium stress, with the main enzymes involved being APX, DHAR, MDHAR, and GR. Figure 4 shows the changes in the activity of GR, APX, DHAR, and MDHAR. Compared with CK, the activities of DHAR and MDHAR in maize leaves treated with CuCd decreased, while the activity of GR and APX increased. The activities of GR, DHAR, MDHAR, and APX in maize leaves treated with NATCA + CuCd were increased compared to CuCd treatment. Compared with CK, the DHAR enzyme activity of Jiuyuan 15 treated with CuCd decreased by 6.36%, 20.52%, 18.55%, 21.71%, and 18.06% in 1–5 days, respectively. The MDHAR enzyme activity decreased by 2.27%, 3.83%, 1.79%, 21.33%, and 27.25%, respectively. Compared with CK, the DHAR enzyme activity of Longfuyu 6 decreased by 1.97%, 13.72%, 14.58%, 35.39%, and 36.42% after 1–5 days of CuCd treatment, respectively. The MDHAR enzyme activity decreased by 4.00%, 9.49%, 21.94%, 35.09%, and 39.80%, respectively. Compared with CuCd, the DHAR enzyme activity of Jiuyuan 15 increased by 5.73%, 20.28%, 13.04%, 67.60%, and 77.98% in the 1–5 days treated with NATCA + CuCd, respectively. The MDHAR enzyme activity increased by 35.78%, 41.10%, 45.49%, 64.75%, and 81.37%, respectively. Compared with CuCd, the DHAR enzyme activity of Longfuyu 6 increased by 34.74%, 14.09%, 53.07%, 94.71%, and 87.53% in NATCA + CuCd treatment for 1–5 days, respectively. The MDHAR enzyme activity increased by 48.26%, 68.69%, 102.09%, 109.20%, and 126.40%, respectively. Compared with CK, the GR enzyme activity of Jiuyuan 15 treated with CuCd increased by 29.27%, 45.27%, 50.20%, and 76.47% in 1–4 days, respectively. The APX enzyme activity increased by 48.46%, 38.51%, 47.58%, and 55.60%, respectively. The GR enzyme activity of Longfuyu 6 increased by 21.25%, 28.33%, 47.12%, and 56.65%, respectively; APX increased by 10.71%, 45.53%, 69.78%, and 82.32%, respectively. Compared with CuCd treatment, the GR enzyme activity of Jiuyuan 15 increased by 11.89%, 16.66%, 20.21%, and 18.45% in the 1–4 days treated with NATCA + CuCd, respectively. The APX enzyme activity increased by 30.36%, 56.07%, 60.98%, and 68.17%, respectively. The GR enzyme activity of Longfuyu 6 increased by 13.07%, 23.15%, 23.21%, and 23.90%, respectively. The APX enzyme activity increased by 30.07%, 54.91%, 52.83%, and 47.50%, respectively (Figure 4).

### 2.5. Content of AsA and GSH

**Figure 5:**
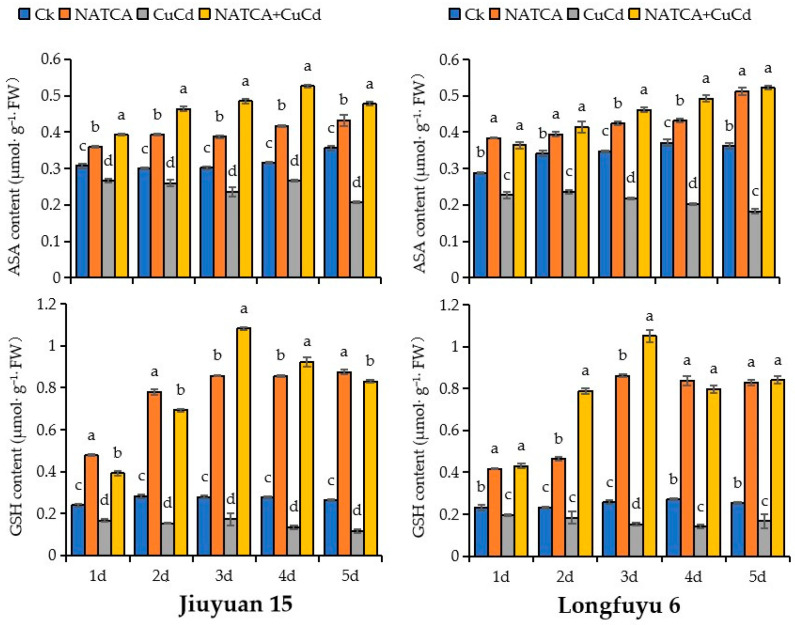
Effects of Folcisteine (NATCA) on the content of ascorbate (AsA) and glutathione (GSH) in maize seedlings leaves under combined copper–cadmium stress. Ck represents 1/2 Hoagland nutrient solution; NATCA represents 1/2 Hoagland nutrient solution containing 20 mg·L^−1^ NATCA; CuCd represents 1/2 Hoagland nutrient solution containing 80 mg·L^−1^ CdCl_2_ and 100 mg·L^−1^ CuSO_4_; NATCA + CuCd represents 1/2 Hoagland nutrient solution containing 20 mg·L^−1^ NATCA, 80 mg·L^−1^ CdCl_2_, and 100 mg·L^−1^ CuSO_4_. Different letters within the same column indicate significant difference at 5% level.

AsA and GSH, as important reducing substances in plants, play a crucial role in preventing membrane lipid peroxidation. Figure 5 illustrates the changes in the content of AsA and GSH. Compared with CK, the AsA content in maize seedling leaves treated with CuCd decreased, and the decrease in Longfuyu 6 was higher than that in Jiuyuan 15. Compared with CuCd treatment, the AsA content in maize seedling leaves increased after NATCA + CuCd treatment, and the increase in Longfuyu 6 was higher than that in Jiuyuan 15. Compared with CK, the AsA content in maize seedling leaves of Jiuyuan 15 decreased by 13.14%, 13.25%, 21.63%, 15.43%, and 42.00% after 1–5 days of CuCd treatment, respectively. The AsA content of Longfuyu 6 decreased by 21.02%, 30.66%, 37.24%, 45.18%, and 49.58%, respectively. Compared with CuCd treatment, the AsA content in maize seedling leaves of Jiuyuan 15 increased by 47.25%, 78.43%, 105.07%, 97.37%, and 130.96% after 1–5 days of NATCA + CuCd treatment, respectively. The AsA content of Longfuyu 6 increased by 59.79%, 75.07%, 111.62%, 142.53%, and 185.76%, respectively.

Compared with CK, the GSH content in maize leaves treated with CuCd decreased. After treatment with NATCA + CuCd, the GSH content in maize leaves increased compared to CuCd treatment. After being treated with CuCd for 1–5 days, the GSH content in the leaves of maize seedlings in Jiuyuan 15 decreased by 30.32%, 45.51%, 37.92%, 51.33%, and 55.77%, respectively, compared to the control group; The GSH content of Longfuyu 6 decreased by 15.40%, 20.98%, 40.78%, 48.47%, and 34.34%, respectively. Compared with CuCd treatment, the GSH content in the leaves of 15 maize seedlings in Jiuyuan increased by 134.73%, 351.18%, 525.62%, 587.61%, and 612.91% after 1–5 days of NATCA + CuCd treatment, respectively. The GSH content of Longfuyu 6 increased by 119.90%, 330.01%, 591.45%, 469.52%, and 405.42%, respectively (Figure 5).

### 2.6. Contents of Endogenous Hormones

In Table 1, the content of IAA, GA, and ZR in maize seedlings decreased, and the content of ABA increased after CuCd treatment. Compared with CK, the IAA content in the leaves of 9-day-15 seedlings treated with CuCd decreased by 4.04%, 14.08%, 31.92%, 38.47%, and 43.05%, respectively. The IAA content in the leaves of Longfuyu maize seedlings decreased by 21.91%, 42.29%, 47.58%, 56.94%, and 57.38%, respectively. Compared with CuCd, the IAA content in maize leaves treated with NATCA + CuCd increased by 37.33%, 34.74%, 57.53%, 45.98%, and 49.37% after 1–5 days, respectively. The leaf content of Longfuyu seedlings increased by 43.83%, 69.27%, 72.24%, 35.47%, and 30.93%, respectively. After CuCd treatment, the GA content of maize seedling leaves in Jiuyuan 15 decreased by 12.21%, 23.13%, 44.61%, 48.28%, and 53.22% from day 1 to day 5 compared to CK. The GA content of Longfuyu 6 seedling leaves decreased by 24.37%, 31.46%, 39.56%, 48.19%, and 56.85%, respectively. Compared with CuCd, the GA content in maize seedling leaves of Jiuyuan 15 increased by 5.21%, 28.18%, 64.14%, 66.40%, and 91.12% after being treated with NATCA + CuCd for 1–5 days, respectively; The GA content in the leaves of Longfuyu 6 seedlings increased by 28.18%, 37.08%, 38.92%, 50.50%, and 70.41%, respectively. Compared with CK, the ZR content in the leaves of 9-day-old seedlings treated with CuCd decreased by 25.19%, 28.55%, 11.70%, 14.10%, and 22.32%, respectively. The ZR content in the leaves of Longfuyu seedlings decreased by 14.45%, 25.16%, 37.30%, 51.72%, and 51.30%, respectively. After treatment with NATCA + CuCd, the ZR content in maize seedling leaves increased by 18.61%, 20.88%, 58.48%, 61.38%, and 44.16% compared to CuCd treatment. The ZR content in Longfuyu seedling leaves increased by 30.75%, 32.24%, 33.28%, and 28.05%, respectively. After CuCd treatment, the ABA content in the leaves of Jiuyuan 15 increased by 17.22%, 46.02%, 59.53%, 60.32%, and 114.58%, respectively, compared to the control group. The ABA content in the leaves of Longfuyu 6 increased by 19.54%, 32.71%, 53.63%, 73.99%, and 50.86%, respectively. Compared with CuCd, after treatment with NATCA + CuCd, the ABA content in the leaves of Jiuyuan 15 maize seedlings decreased by 9.29%, 12.18%, 9.97%, 24.85%, and 26.23%, respectively. The ABA content in Longfuyu 6 maize seedlings decreased by 6.92%, 14.06%, 13.85%, 10.89%, and 22.80%, respectively (Table 1).

### 2.7. Balance of Endogenous Hormones

**Figure 6:**
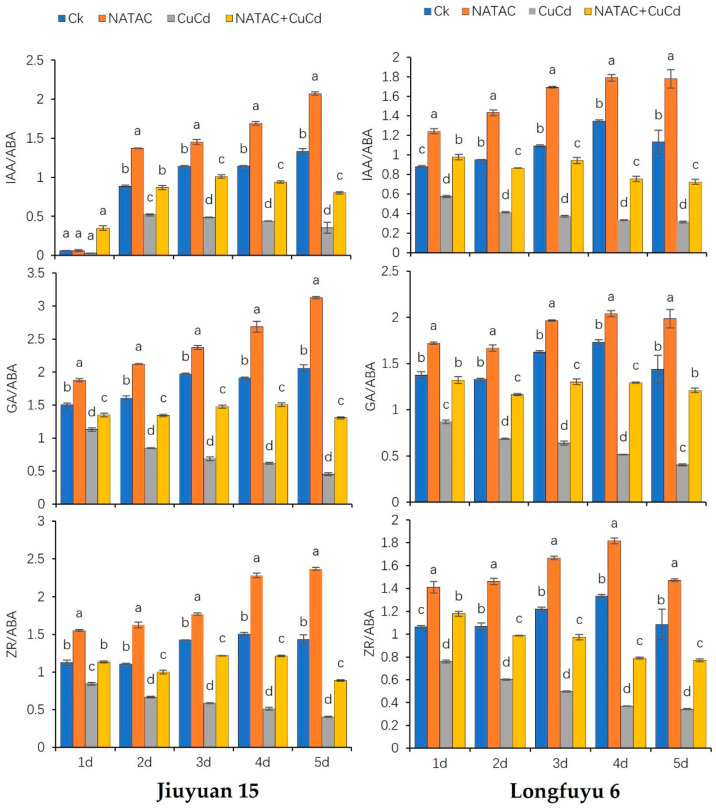
Effects of Folcisteine (NATCA) on maize seedlings’ endogenous hormones balance (IAA/ABA, GA/ABA, and ZR/ABA) under combined copper–cadmium stress. Ck represents 1/2 Hoagland nutrient solution; NATCA represents 1/2 Hoagland nutrient solution containing 20 mg·L^−1^ NATCA; CuCd represents 1/2 Hoagland nutrient solution containing 80 mg·L^−1^ CdCl_2_ and 100 mg·L^−1^ CuSO_4_; NATCA + CuCd represents 1/2 Hoagland nutrient solution containing 20 mg·L^−1^ NATCA, 80 mg·L^−1^ CdCl_2_, and 100 mg·L^−1^ CuSO_4_. IAA represents auxin; GA represents gibberellin; ZR represents zeatin nucleoside; ABA represents abscisic acid. Different letters within the same column indicate significant difference at 5% level.

As shown in Figure 6, the ratios of IAA, GA, ZR, and ABA in maize seedling leaves decreased after CuCd treatment, and the decrease in Longfuyu 6 was higher than that in Jiuyuan 15. After treatment with NATCA + CuCd, the levels of IAA/ABA, ZR/ABA, and GA/ABA in maize seedling leaves showed an increasing trend compared to CuCd treatment. Compared with CK, after CuCd treatment, the IAA/ABA levels in the leaves of Jiuyuan 15 seedlings decreased by 50.73%, 41.12%, 57.32%, 61.62%, and 73.48% in 1–5 days, respectively. The IAA/ABA of Longfuyu 6 leaves decreased by 34.67%, 56.52%, 65.86%, 75.25%, and 72.31%, respectively. After treatment with NATCA + CuCd, the IAA/ABA levels in the leaves of 15 maize seedlings in Jiuyuan increased by 1042.29%, 66.83%, 106.78%, 114.23%, and 126.84% compared to CuCd levels within 1–5 days. The IAA/ABA of Longfuyu 6 leaves increased by 70.42%, 109.69%, 152.77%, 125.93%, and 130.65%, respectively, from day 1 to day 5. After 1–5 days of CuCd treatment, the GA/ABA levels in the leaves of Jiuyuan 15 seedlings decreased by 25.03%, 47.35%, 65.27%, 67.74%, and 78.20%, respectively. The leaves of Longfuyu 6 decreased by 36.74%, 48.36%, 60.60%, 70.22%, and 71.96%, respectively. After NATCA + CuCd treatment, Jiuyuan 15 showed an increase of 19.31%, 58.62%, 115.37%, 144.18%, and 190.19% compared to CuCd treatment from 1 to 5 days. Longfuyu 6 showed an increase of 51.83%, 69.81%, 103.66%, 150.96%, and 199.94% after 1–5 days, respectively. Compared with CK, after CuCd treatment, the ZR/ABA of Jiuyuan 15 leaves decreased by 25.09%, 39.73%, 58.84%, 65.91%, and 71.68% in 1–5 days, respectively. The ZR/ABA of Longfuyu 6 decreased by 28.43%, 43.63%, 59.17%, 72.26%, and 68.46%, respectively. Compared with CuCd, after NATCA + CuCd treatment, the ZR/ABA of Jiuyuan15 increased by 34.48%, 49.61%, 107.95%, 136.70%, and 118.94% in 1–5 days, respectively. Longfuyu 6 increased by 54.88%, 63.81%, 95.58%, 113.54%, and 125.09%, respectively (Figure 6).

## 3. Discussion

Folcisteine (NATCA) is often used as an intermediate in pesticides and mixed with other substances to play a role in agricultural production. A plant growth regulator combination synthesized from hemiphyllin, inositol peptide, and potassium magnesium sulfate has specific functions of stimulating plant absorption of water and fertilizer, stabilizing the structure of large molecular substances, increasing the activity of metabolic enzymes in plants, regulating redox potential, increasing amino acid content in plants, and improving protein synthesis rate [57]. A 1:1 plant growth regulator composed of hemifolin and auxin can act on agricultural production in the form of powder solubilizer, particle solubilizer, and ultra-low molecular spray, which is conducive to promoting fruit expansion and grain plumpness after passing away, improving root growth capacity and increasing yield [58]. A plant growth regulator combination composed of hemiphyllin and sodium salicylate can effectively improve the growth of crops such as wheat, rice, maize, soybeans, and peanuts under stress environments, enhance crop salt and alkali tolerance, and alleviate cell damage under crop stress [59]. Research has found that NATCA increases the height, crown length, and volume of apricot trees, and improves fruit setting and retention rates. Applying 100 ppm NATCA increases tree height, crown length, volume, fruit setting, and retention rates by 32.54%, 61.37%, 30.13%, 34.26%, and 50.97%, respectively [61,62]; the yield was increased by applying 100 ppm NATCA, resulting in an increase of 14.59%, 12.00%, 28.82%, 27.12%, and 35.66% in fruit length, diameter, weight, volume, and yield, respectively [63]. The lowest fruit drop rate was observed when NATCA was applied to apricot trees during the petal shedding period, with a drop rate of only 27.03% when 50 ppm NATCA was applied [64]. Spraying NATCA on the leaves during the flowering period of apricot trees increased chlorophyll content. Spraying NATCA on the leaves during the flower bud period of apricot trees increased the total sugar, reducing sugar, and non-reducing sugar content of the fruit. When 100 ppm NATCA was applied, the total sugar content increased by 30.14% compared to the control, the reducing sugar content increased by 19.15%, and the non-reducing sugar content increased by 21.06%. Hemiphyllin increased the sugar content of the fruit by regulating the balance of endogenous hormones and amino acid content [65]. Research has found that hemiphyllin can promote the growth of watermelon, tomato, and rice seedlings, and facilitate the accumulation of dry matter in crops. Treatment with hemiphyllin significantly increases both fresh and dry weight of crops [60].

At high concentrations, all heavy metals accumulate in plant tissues over time, causing toxicity and inhibiting plant growth, with Cd and Cu being the most commonly reported toxic heavy metals [66]. The main carboxylase, 1,5-diphosphoribulose carboxylase/oxygenase (Rubisco), is used by all photosynthetic organisms to fix atmospheric carbon dioxide [67]. After CuCd stress, the activity of RUBP carboxylase in maize seedlings weakened, indicating that CuCd stress not only destroyed the structure of chloroplasts but also hindered the C3 pathway of maize photosynthesis. Research has shown that stress from two heavy metals (Cd, Cu) significantly reduces tobacco growth, total chlorophyll content, Rubisco content, and activity [28]. Phosphoenolpyruvate carboxylase (PEPCase) is a cytoplasmic enzyme commonly found in higher plants [68]. The leaves of C4 and Sedum acid metabolizing plants contain high levels of PEPC, which catalyze initial CO_2_ fixation during photosynthesis [69]. This study found that CuCd stress reduced the activity of PEPC enzyme and hindered the C4 pathway. Cd stress reduces the activity of RUBP carboxylase and PEPC enzyme in maize [70]. The C3 and C4 pathways are pathways for maize carbon assimilation. CuCd stress hinders the carbon assimilation process in maize and inhibits its photosynthesis. Cadmium stress can reduce the activity of the carboxylation system in maize, hinder the conversion of light energy to chemical energy, and accumulate a large amount of excess electrons at PSI. The coordination between PSII and PSI decreases, leading to a decrease in net photosynthetic rate [71]. Research has found that using pig manure with high copper concentration on maize can inhibit the photosynthetic parameters of maize, significantly reducing the net photosynthetic rate, intercellular CO_2_ concentration, transpiration rate, and photosynthetic pigment content of maize leaves [72]. This study indicates that the application of NATCA to maize seedlings significantly increases the activities of RUBP carboxylase and PEPC enzyme, enhances carbon assimilation efficiency, and increases photosynthetic rate. Exogenous substances can effectively alleviate the damage of Cd to photosynthesis, enhance the light capture ability of seedling leaves, promote photosynthesis, and enhance the resistance of maize seedlings to Cd stress [73]. This study showed that applying NATCA to maize under CuCd stress increased the activity of RUBP and PEPC enzymes, restored normal carbon assimilation pathways, and resulted in healthy oil green leaves, indicating that chlorophyll was also protected and photosynthesis was greatly improved.

When plants are subjected to CuCd stress, they produce a large amount of reactive oxygen species. When there is a serious imbalance between the production of reactive oxygen species and antioxidant defense, oxidative stress occurs [74,75]. The main sites of ROS damage are mitochondria, chloroplasts, peroxisomes, plasma membranes, and extracellular vesicles. Due to the limitations of stress conditions, the inhibition of CO_2_ fixation can lead to a reduction in carbon fixation and a decrease in the oxidation of electron acceptors (NADP) in photosynthesis [76]. This study indicates that under CuCd stress, the content of O_2_·^−^ and H_2_O_2_ in maize seedling leaves increases, and the damage area of reactive oxygen species to leaves increases. The appearance of brown spots (H_2_O_2_) and dark blue spots (O_2_·^−^) on plant leaves under cadmium stress is very obvious [77]. Under Cd stress, the accumulation of O_2_·^−^ and H_2_O_2_ in wheat seedlings may be disrupted by excessive H_2_O_2_, which may damage antioxidant enzyme activity. Unresolved reactive oxygen species spontaneously react with organic molecules, leading to membrane lipid peroxidation, protein oxidation, enzyme inhibition, as well as DNA and RNA [78]. The production of reactive oxygen species (ROS) is a continuous source of attack on genetic material, and nutrients, hormones, exogenous substances, and environmental influences can enhance or partially reduce ROS. Previous studies have found that Cd entering cells increases the content of Cu [79]. Cd itself increases the content of O_2_·^−^ and H_2_O_2_ in the cell. After the increase in Cu content, the production of HO^−^ in H_2_O_2_ is increased through the Fenton reaction. HO^−^ free radicals induce single and double strand breaks in DNA, causing DNA damage, which may be caused by the reaction between HO^−^ free radicals and hydrogen atoms on the carbon C_3_, C_4_, C_5,_ or C2 of 2-deoxyribose residues [80,81,82,83]. This study indicates that under CuCd stress, the permeability of cell membranes increases, and the electrolyte leakage rate of maize increases, resulting in plant dehydration. Previous studies have shown that under the combined stress of copper and cadmium, the electrical conductivity of cattail increases, and membrane lipids are damaged [84]. Cd leads to the accumulation of H_2_O_2_, an increased degree of lipid peroxidation, and elevated electrical conductivity [85]. The toxicity of Cu increased the production of hydrogen peroxide (H_2_O_2_), malondialdehyde (MDA), and electrolyte leakage in the tissues of European rapeseed leaves and roots [86]. This study indicates that NATCA can alleviate the oxidative stress response of maize seedlings to CuCd stress and reduce the damage of reactive oxygen species to maize seedlings. Research has shown that exogenous proline alleviates oxidative damage caused by Cd accumulation, and the application of proline to olives under cadmium stress increases antioxidant enzyme activity, photosynthetic activity, nutritional status, plant growth, and olive fruit oil content. In this study, no significant differences in reactive oxygen species were found between maize seedlings treated with NATCA alone and those not treated with NATCA [87].

Maize seedlings possess a relatively strong ability to accumulate cadmium and exhibit tolerance to high concentrations of cadmium. Under cadmium (Cd) stress, oxidative damage constitutes the primary cause of cellular damage. This oxidative damage occurs as a result of alterations in membrane permeability, which subsequently triggers the production of reactive oxygen species (ROS) at the organelle level, thereby impeding plant growth [88]. To counteract oxidative stress, plants have evolved diverse defense strategies aimed at safeguarding themselves against the detrimental effects of ROS. These defense strategies primarily encompass antioxidant enzymes, such as guaiacol peroxidase (POD), superoxide dismutase (SOD), ascorbate peroxidase (APX), catalase (CAT), and glutathione reductase (GR). Additionally, non-enzymatic antioxidants like ascorbic acid (AA), glutathione (GSH), tocopherols, and carotenoids also play significant roles in this regard [89].

It has been observed that variations in soil cadmium concentration have a direct impact on certain physiological parameters in maize seedling leaves. Specifically, a decrease in soil cadmium concentration leads to a reduction in the content of glutathione (GSH), while an increase in soil cadmium concentration results in enhanced activities of guaiacol peroxidase (POD), superoxide dismutase (SOD), catalase (CAT), and ascorbate peroxidase (APX) [90]. Moreover, research has demonstrated that in barley, which is sensitive to Cd, treatment with Cd leads to an elevation in the levels of ascorbic acid, particularly in non-protein sulfhydryl (SH) groups [91]. Osmotic substances also contribute to plants’ ability to resist copper (Cu) and cadmium (Cd) stress [92]. Proline (Pro) is involved in multiple aspects of plant physiology and development. It not only functions as a protein amino acid but also serves as an important osmoregulatory substance, playing a role in stress resistance. Proline is capable of scavenging reactive oxygen species (ROS), thereby stabilizing proteins [93]. Furthermore, changes in proline metabolism can influence the cellular redox state, prompting corresponding metabolic adjustments. Additionally, proline helps to buffer the cytoplasmic pH value and can act as a source of carbon and nitrogen to support plant growth following the alleviation of stress [94,95].

The good coordination between APX, MDHAR, DHAR, and GR in the AsA–GSH cycle can also increase plant tolerance to Cu, Cd, or any other metal toxicity [96]. This study indicates that after CuCd stress, the activities of MDHAR and DHAR in leaves decrease, while the activities of APX and GR increase. DHAR participates in the process of reducing DHA to AsA. MDHAR plays a crucial role in preventing the reduction in AsA and ascorbic acid redox buffer under stress conditions. Previous studies have shown that Cd significantly reduces the activity of DHAR and MDHAR, respectively [97]. Research has found that the activity of APX increases after Cd + As treatment, which may be due to the increase in H_2_O_2_ content [98]. Research has shown that after Cd treatment, the GR content in wheat leaves increases, and the upregulation of GR activity comes from modifications during translation [99]. AsA and GSH can reduce the accumulation of reactive oxygen species in chloroplasts, and when AsA and GSH levels are high, they can protect cells from oxidative damage caused by Cu and Cd stress. The GSH content of wheat seedlings decreased under copper–cadmium composite stress [100]. Therefore, the decrease in AsA content in seedlings affected by Cd in this study confirms the decrease in MDHAR and DHAR. Many previous studies have shown that the application of exogenous plant growth regulators can enhance the ability of the AsA–GSH cycle to clear reactive oxygen species. Plant growth regulators (2,4-brassinosteroids) can improve grape resistance to Cu and enhance antioxidant capacity [101]. Our study indicates that the application of NATCA under CuCd stress can effectively increase the activity of APX, MDHAR, DHAR, and GR, as well as the content of AsA and GSH.

Abscisic acid, ethylene, etc., are considered stress response hormones; auxins, cytokinins, and other hormones belong to the category of growth-promoting hormones [102]. This study showed that under CuCd stress, the content of IAA, GA, and ZR in maize seedlings decreased, while the content of ABA increased, indicating that CuCd stress inhibited the production of growth promoting hormones in maize seedlings and activated stress hormones to resist CuCd stress. Previous studies have found that the IAA content in maize root tips significantly decreases under Cu stress [103]. IAA and GA3 play a protective role under copper stress by restoring redox homeostasis, thereby reducing protein damage in roots [104]. Research has shown that under high concentration cadmium treatment, the content of IAA, GA, and ZR decreases, while the content of ABA increases [105]. Research has shown that the GA content in tobacco roots decreases under cadmium stress [106]. GA can promote the increase in precursors for IAA synthesis and facilitate the transformation of IAA from bound to free form. As a result, GA content gradually decreases, leading to a gradual decrease in IAA content [107]. The cell wall of pea roots seems to increase the ability of peroxidase activity, which oxidizes IAA in the presence of cadmium and copper ions. This reduces endogenous auxin in plant roots under metal stress, thereby inhibiting the growth of pea seedlings [108]. Under Cu and Cd stress, IAA plants can trigger short-term responses (such as closing stomata) to resist the damage caused by Cu and Cd. After the accumulation of Cu and Cd, the concentrated genes that produce ABA are upregulated, such as the maize xanthin cyclooxygenase gene (ZEP), aldehyde oxidase gene (AAO3), dioxygenase gene (NCED3), and molybdenum cofactor sulfurase gene (MCSU) [109]. Under cadmium stress, ABA can lead to oxidative stress, and the enhancement of oxidative stress tolerance may mean that plants need to mobilize their entire antioxidant defense system, including enzymatic and non-enzymatic components, to resist oxidative damage to stressed plant tissues, rather than some enzymes or metabolites [110,111]. ABA enhances plant tolerance to cadmium stress and reduces absorption of heavy metals, indicating that ABA may be involved in signaling events leading to reduced Cd accumulation or activation of other defense mechanisms against heavy metal stress [112]. This study found that after CuCd treatment, the balance of endogenous hormones was disrupted, and the values of IAA/ABA, GA/ABA, and ZR/ABA decreased compared to the control. Under Cd stress, the ABA/Zr ratio in the leaf tissue of rapeseed significantly increased [48]. High concentrations of cadmium stress caused a decrease in the content of ZR, IAA, and GA in Sedum erinaceus, while the content of ABA significantly increased [113]. The values of ABA/IAA, ABA/GA, and ABA/ZR increase with the increase of cadmium concentration. Cadmium has an inhibitory effect on IAA, ZR, and GA, and a stimulating effect on ABA synthesis [114]. Plant growth regulators can increase the content of plant growth promoting hormones and maintain the balance of endogenous hormones in the plant body. Research has found that exogenous CPPU (cytokinin plant growth regulator) can inhibit the absorption and transport of Cu and Cd by cells [115]. Research has found that CPPU increases the content of IAA, GA3, and ZR in rice husks, while reducing the content of ABA [116]. This study shows that applying NATCA to maize seedlings under CuCd stress reduces growth promoting hormones and stress response hormones, while maintaining hormone balance. This indicates that exogenous NATCA can promote maize seedling growth by regulating hormone levels, improve maize seedlings’ resistance to CuCd, and alleviate the damage caused by maize seedlings in CuCd environment. Copper cadmium composite stress has significant effects on multiple aspects of maize growth, including growth and development, physiological and biochemical characteristics, mineral nutrient absorption, and quality. Although some progress has been made in mitigating measures, further in-depth research is still needed on the mechanism of copper–cadmium composite stress, especially at the molecular level, to explore the key genes and signaling pathways that maize responds to composite stress, in order to more accurately select tolerant varieties, develop more effective remediation and prevention strategies, ensure the safe production of maize in polluted soil environments, and safeguard food security and ecological environment health.

## 4. Materials and Methods

### 4.1. Plant Materials and Growth Condition

Based on preliminary experiments, Jiuyuan 15 (tolerant to CuCd stress) and Longfuyu 6 (intolerant to CuCd stress) were selected as materials. Half leaf extract (NATCA, Folcisteine) was purchased from Zhengzhou Zhengshi Chemical Products Co., Ltd.(Zhengzhou City, Henan Province, China), CAS number: 5025-82-1, molecular formula C_6_H_9_NO_3_S, molecular weight 175.2056. Copper stress was treated with copper sulfate pentahydrate, molecular formula CuSO_4_·5H_2_O, molecular weight: 249.685, CAS number: 7758-99-8. Cadmium stress is treated with anhydrous cadmium chloride, molecular formula CdCl_2_, molecular weight: 183.32, CAS number: 10108-64-2. We select healthy and plump seeds, disinfect them with 10% sodium hypochlorite solution, wash and soak for 10 h, evenly spread them into the germination box, and transfer them to a constant temperature incubator (26 ± 1 °C). After the seeds germinate and the embryo grows to 1.5–2 cm, select germinated seeds with consistent growth and plant them in a non-light culture pot containing 20 L of 1/2 Hoagland nutrient solution (pH = 6.0). Change the nutrient solution once in 3D, ventilate the air pump at regular intervals (45 min·h^−1^), expose to light for 12 h, and maintain a temperature of 28 ± 1 °C. When the seedlings grow to three leaves and one center, select seedlings with consistent growth for treatment. Each group has 60 seedlings, divided into four groups. The experimental processing is as follows:

(1) Control group (CK), 1/2 Hoagland nutrient solution;
(2) NATCA treatment (NATCA), add 20 mg·L^−1^ NATCA to 1/2 Hoagland nutrient solution;
(3) Copper and cadmium stress treatment (CuCd), adding 80 mg·L^−1^ CdCl_2_ and 100 mg·L^−1^ CuSO_4_ to 1/2 Hoagland nutrient solution [117,118]; and
(4) Copper and cadmium stress combined with NATCA treatment (NATCA + CuCd), 20 mg·L^−1^ NATCA, 80 mg·L^−1^ CdCl_2_, and 100 mg·L^−1^ CuSO_4_ were added to 1/2 Hoagland nutrient solution.

Add 80 mg·L^−1^ CdCl_2_ and 100 mg·L^−1^ CuSO_4_ to the three leaves at the same time. The seedlings that require CuCd treatment should be added to the copper–cadmium treatment solution twice, with an interval of 12 h, to provide a buffer time for the seedlings. Seedlings that require NATCA treatment should be treated with copper–cadmium compound for 12 h before adding NATCA. Adjust pH once a day, with a photoperiod of 12/12 (day/night), temperature of (28 ±1) °C/(25 ± 1) °C day/night, light intensity of around 400 μ mol·m^−2^·s^−1^, and relative humidity of 60% to 70%. The 12th hour after adding various treatments is taken as the 1st day of sampling.

### 4.2. Determination of RuBPCase (Ribulose-1,5-Bisphosphate Carboxylase) and PEPCase (Phosphoenolpyruvate Carboxylase)

Samples were taken at regular intervals within 1–5 days after treatment, and seedlings with consistent growth were selected. The leaves were cut and ground into a homogenate at 2–8 °C in PBS (pH = 7.4). Take the supernatant after 20 min (3000 r·min^−1^). Use the RUBP carboxylase detection kit and PEPCase ELISA kit provided by Nanjing Jiancheng Bioengineering Institute (Nanjing City, China) for detection on 5 January 2022 (http://www.njjcbio.com/). After the color development step is terminated, measure the absorbance of each well using a spectrophotometer at a wavelength of 450 nm and repeat three times.

### 4.3. Determination of O_2_·^−^ and H_2_O_2_

On the 5th day of processing, take the third leaf, wash it, and dry it with filter paper. Repeat each process three times. NBT staining method is used for measure O_2_·^−^. We select the seedling leaves in a 1 mg·L^−1^ NBT solution, evacuate and place in a dark place for 10 h, then boil with anhydrous ethanol to remove green. H_2_O_2_ was stained using the DAB staining method. Place the seedling leaves in 2 mg·L^−1^ DAB solution, vacuum, and place in the dark for 10 h. Boil with anhydrous ethanol to remove the green color. After decolorization, select the leaves with obvious staining and take photos [119,120].

### 4.4. Determination of Electrolyte Leakage (EL)

On the 5th day after treatment, 1 g of leaves was taken from each group, washed with deionized water, dried with filter paper, and then decomposed into small square pieces of one square centimeter. Soak completely in 10 mL of deionized water for 24 h. Measure the initial conductivity (EC1) using a Thunderbolt DDS-11A conductivity meter. Boil water for 10 min, cool down, and measure the final conductivity (EC2). Calculate the electrolyte leakage (EL) as EC1/EC2 × 100 [121].

### 4.5. Determination of Glutathione Reductase Activity (GR)

Take maize seedlings with consistent growth from each treatment at regular intervals every day for 1–5 days after treatment, and separate the roots and leaves. Cut each sample into pieces, add PBS (pH = 7.4), grind to a homogenate at 2–8 °C, and leave for 20 min (3000 r/min) to obtain the supernatant. The GR activity was measured using the glutathione reductase activity detection kit provided by Nanjing Jiancheng Bioengineering Institute (http://www.njjcbio.com/) and assessed on 5 January 2022. Measure the absorbance using a spectrophotometer (450 nm) within 15 min after adding the stop solution.

### 4.6. Determination of Ascorbate Peroxidase Activity (APX)

Take maize seedlings with consistent growth from each treatment at regular intervals every day for 1–5 days after treatment. Add 5 mL of pre-cooled extract and quartz sand to the roots and leaves, respectively, and centrifuge at 12,000× *g*·min^−1^ for 20 min. Take the supernatant as the crude extract. Add 2.9 mL of APX reaction solution to 0.1 mL of crude extract, and then compare the color [122].

### 4.7. Determination of Dehydroascorbate Reductase Activity (DHAR)

The preliminary preparation work is the same as the steps for GR activity determination. DHAR was detected using the dehydroascorbate reductase (DHAR) activity detection kit provided by Nanjing Jiancheng Bioengineering Institute (http://www.njjcbio.com/) and assessed on 5 January 2022. Measure the absorbance using a spectrophotometer.

### 4.8. Determination of Monodehydroascorbate Reductase Activity (MDHAR)

The preliminary preparation work is the same as the steps for GR activity determination. The MDHAR was detected using the MDHAR detection kit from Nanjing Jiancheng Bioengineering Research Institute (http://www.njjcbio.com/) and assessed on 5 January 2022. Measure the absorbance using a spectrophotometer.

### 4.9. Determination of Ascorbate (AsA) Content

Take maize seedlings with consistent growth from each treatment at regular intervals every day for 1–5 days after treatment. Grind 0.5 g of the sample with 5% hydrochloric acid, centrifuge at 15,000× *g* for 20 min, and take the supernatant as the crude extract. Then, add PBS and water to the supernatant (supernatant: 150 MmPBS: water = 1:1:1). Vortex the mixture and let it stand at room temperature for 1 min. Add 10% TCA, 45% H_3_PO_4_, and 3% FeCl_3_ for vortexing. Insulate at 37 °C for 1 h. Use a spectrophotometer for measurement [123].

### 4.10. Determination of Glutathione (GSH) Content

Prepare the crude extract as shown in the AsA content determination, take the supernatant to measure the total content of GSH and GSSG, add the crude enzyme solution to the reaction solution, and then perform colorimetric analysis. Measure the content of GSSG again, add the crude enzyme solution diluted 50 times to the pyrimidine water bath, and perform colorimetric analysis. Subtracting the two gives the GSH content [124].

### 4.11. Determination of Endogenous Hormones Content

Samples were taken at 24, 48, 72, 96, and 108 h after processing for the determination of endogenous hormones. Simultaneous determination of IAA, GR, ZR, and ABA using high-performance liquid chromatography. Add 80% methanol prepared with 1 mmol·L^−1^ BHT to the ground seedling leaves under ice bath conditions to obtain the sample extract. After centrifugation, the supernatant was passed through a C-18 solid-phase extraction column, and methanol was removed. The enzyme-linked immunosorbent assay kit and Bio TEK Elx-800 fully automatic enzyme-linked immunosorbent assay [125].

### 4.12. Data Analysis

The data were expressed by the measured mean value, analyzed by SPSS19.0 (IBM SPSS Statistics, 2010), and compared by Duncan’s new complex difference method (α = 0.05), and origin 8 is used for drawing.

## 5. Conclusions

Employing exogenous substances to bolster maize’s resistance to copper and cadmium stress represents an efficient and eco-friendly development strategy. Research indicates that the application of exogenous NATCA boosts the activities of key photosynthetic enzymes RUBPCase and PEPCase, thereby enhancing photosynthetic capacity. Simultaneously, the reduction in O_2_·^−^ and H_2_O_2_ levels mitigates the damage to leaf cells inflicted by reactive oxygen species. The introduction of exogenous NATCA elevates the activities of MDHAR and DHAR, antioxidants within the ascorbate–glutathione cycle in maize seedling leaves, and increases the concentrations of non-enzymatic antioxidants AsA and GSH. Consequently, this enhances the leaves’ capacity to scavenge reactive oxygen species via the ascorbate–glutathione cycle, further reducing the harm inflicted by copper and cadmium combined stress on maize seedlings. Upon the application of exogenous NATCA, the concentrations of IAA, GA, and ZR rise, whereas ABA levels decline. NATCA fosters seedling growth by modulating the content and balance of endogenous hormones, thereby augmenting maize’s tolerance to copper and cadmium combined stress. This study contributes to advancing the application of exogenous plant growth regulators in environmental remediation. In the future, NATCA can be developed into new types of agricultural products such as fertilizers, plant growth regulators, or soil conditioners, which can alleviate the toxic effects of heavy metals on maize, promote maize growth, enhance the stress resistance of maize, and thus ensure the yield and quality of grain. Due to the differences between hydroponic culture and actual soil environments, the results of this experiment on the physiological and biochemical changes and tolerance of plants under heavy metal stress have considerable uncertainty when extrapolated to field conditions. Further verification of the field is needed in the future.

## Figures and Tables

**Table 1:** Effects of Folcisteine (NATCA) on maize seedlings’ endogenous hormones content under combined copper–cadmium stress.

Parameters	Variety	Treatment	1d	2d	3d	4d
IAA (ng·g^−1^·FW)	Jiuyuan15	Ck	159.64 ± 0.20 c	172.18 ± 4.11 c	220.10 ± 2.36 c	232.47 ± 1.54 b
		NATCA	230.22 ± 2.87 a	234.30 ± 2.54 a	251.78 ± 6.68 a	258.56 ± 4.20 a
		CuCd	153.19 ± 1.58 d	147.94 ± 2.11 d	149.85 ± 1.35 d	143.03 ± 1.57 d
		NATCA + CuCd	210.38 ± 1.75 b	199.33 ± 2.50 b	236.07 ± 3.49 b	208.81 ± 2.01 c
	Longfuyu 6	Ck	208.45 ± 2.09 c	242.94 ± 1.68 b	252.68 ± 2.76 b	294.48 ± 2.39 b
		NATCA	274.22 ± 2.64 a	314.91 ± 2.60 a	337.72 ± 1.92 a	348.69 ± 2.31 a
		CuCd	162.78 ± 0.86 d	140.21 ± 2.91 d	132.45 ± 1.28 d	126.80 ± 1.85 d
		NATCA + CuCd	234.12 ± 3.41 b	237.32 ± 2.46 c	228.14 ± 4.39 c	171.77 ± 5.73 c
GA (ng·g^−1^·FW)	Jiuyuan15	Ck	314.01 ± 9.62 b	312.76 ± 5.19 b	379.47 ± 2.09 b	388.38 ± 2.68 b
		NATCA	354.85 ± 6.15 a	361.99 ± 3.38 a	410.63 ± 7.68 a	409.78 ± 7.92 a
		CuCd	275.66 ± 2.17 c	240.41 ± 1.59 c	210.19 ± 7.29 d	200.86 ± 4.24 d
		NATCA + CuCd	290.03 ± 4.29 c	308.17 ± 3.31 b	345.00 ± 3.63 c	334.23 ± 5.39 c
	Longfuyu 6	Ck	326.12 ± 6.27 b	339.96 ± 1.25 b	375.61 ± 0.95 b	378.64 ± 4.13 b
		NATCA	379.67 ± 1.89 a	366.66 ± 4.46 a	392.11 ± 2.48 a	397.22 ± 3.45 a
		CuCd	246.64 ± 3.06 c	233.02 ± 1.48 d	227.02 ± 2.50 d	196.18 ± 2.33 d
		NATCA + CuCd	316.14 ± 5.29 b	319.43 ± 2.43 c	315.37 ± 0.49 c	295.26 ± 1.53 c
ZR (ng·g^−1^·FW)	Jiuyuan15	Ck	234.31 ± 2.57 c	215.62 ± 1.39 c	274.41 ± 1.43 c	305.31 ± 4.45 b
		NATCA	293.33 ± 3.39 a	277.17 ± 4.10 a	306.52 ± 3.23 a	348.35 ± 0.68 a
		CuCd	205.85 ± 1.96 d	189.69 ± 1.68 d	180.16 ± 1.24 d	166.81 ± 5.30 d
		NATCA + CuCd	244.15 ± 1.98 b	229.30 ± 1.71 b	285.51 ± 2.74 b	269.19 ± 1.19 c
	Longfuyu 6	Ck	252.01 ± 1.64 c	273.26 ± 5.39 b	282.17 ± 1.34 b	291.42 ± 2.88 b
		NATCA	311.39 ± 7.83 a	321.64 ± 3.43 a	332.65 ± 3.46 a	354.14 ± 1.98 a
		CuCd	215.59 ± 0.95 d	204.52 ± 1.27 d	176.93 ± 2.02 d	140.68 ± 1.69 d
		NATCA + CuCd	281.88 ± 1.32 b	270.46 ± 1.00 c	235.82 ± 2.04 c	180.14 ± 1.37 c
ABA (ng·g^−1^·FW)	Jiuyuan15	Ck	208.45 ± 5.03 b	194.55 ± 2.10 c	192.54 ± 0.82 c	203.17 ± 0.37 c
		NATCA	189.07 ± 1.68 c	170.85 ± 1.85 d	173.35 ± 1.23 d	152.68 ± 2.30 d
		CuCd	244.34 ± 5.01 a	284.09 ± 4.35 a	307.16 ± 1.53 a	325.72 ± 2.00 a
		NATCA + CuCd	215.34 ± 1.58 b	229.62 ± 4.48 b	234.06 ± 2.03 b	222.01 ± 1.35 b
	Longfuyu 6	Ck	237.26 ± 2.69 b	256.10 ± 2.24 c	231.58 ± 2.17 b	218.77 ± 0.97 c
		NATCA	220.83 ± 2.54 c	220.08 ± 3.80 d	199.51 ± 0.41 c	194.95 ± 2.41 d
		CuCd	283.62 ± 3.18 a	339.86 ± 1.20 a	355.76 ± 8.27 a	380.63 ± 1.62 a
		NATCA + CuCd	239.50 ± 3.90 b	274.36 ± 2.02 b	242.58 ± 6.05 b	228.28 ± 1.06 b

Note: Data are expressed as mean ± standard deviation. Different letters within the same column indicate significant difference at 5% level. Ck represents 1/2 Hoagland nutrient solution; NATCA represents 1/2 Hoagland nutrient solution containing 20 mg·L^−1^ NATCA; CuCd represents 1/2 Hoagland nutrient solution containing 80 mg·L^−1^ CdCl_2_ and 100 mg·L^−1^ CuSO_4_; NATCA + CuCd represents 1/2 Hoagland nutrient solution containing 20 mg·L^−1^ NATCA, 80 mg·L^−1^ CdCl_2_, and 100 mg·L^−1^ CuSO_4_. IAA represents auxin; GA represents gibberellin; ZR represents zeatin nucleoside; ABA represents abscisic acid. Different letters within the same column indicate significant difference at 5% level.

## Data Availability

The data presented in this study are available upon request from the corresponding author.

## Abbreviations

The following abbreviations are used in this manuscript:

NATCA	folcisteine
Cu	copper
Cd	cadmium
ROS	reactive oxygen species
RUBPCase	ribulose-1,5-bisphosphate carboxylase
PEPCase	phosphoenolpyruvate carboxylase
AsA	ascorbate
GSH	glutathione
IAA	auxin
GA	gibberellin
ZR	zeatin nucleoside
ABA	abscisic acid.
EL	electrolyte leakage
GR	glutathione reductase activity
APX	ascorbate peroxidase activity
DHAR	dehydroascorbate reductase activity
MDHAR	monodehydroascorbate reductase activity
O_2_^−^	superoxide anion
H_2_O_2_	hydrogen peroxide
